# Editorial: Molecular Basis of Stage Conversion in Apicomplexan Parasites

**DOI:** 10.3389/fcimb.2021.680184

**Published:** 2021-04-16

**Authors:** Mathieu Gissot, Mattie C. Pawlowic, Katarzyna K. Modrzynska, Maria E. Francia

**Affiliations:** ^1^ Univ. Lille, CNRS, Inserm, CHU Lille, Institut Pasteur de Lille, U1019 - UMR 9017 - CIIL - Center for Infection and Immunity of Lille, Lille, France; ^2^ Welcome Centre for Anti-Infectives Research, School of Life Sciences, University of Dundee, Dundee, United Kingdom; ^3^ Welcome Centre for Integrative Parasitology, University of Glasgow, Glasgow, United Kingdom; ^4^ Laboratory of Apicomplexan Biology, Institut Pasteur de Montevideo, Montevideo, Uruguay; ^5^ Department of Parasitology and Mycology, School of Medicine, Universidad de la República, Montevideo, Uruguay

**Keywords:** Apicomplexa, *Toxoplasma*, *Plasmodium*, *Cryptosporidium* spp., Eimeria, parasite, differentiation

## Introduction

The Apicomplexa is a large phylum of intracellular parasitic protozoa with more than 6000 species that present a set of invasion organelles organized in an apical complex (Adl et al., 2007). It is estimated that every metazoan can be infected by at least one species from this group (Morrison, 2009), indicating their diversity and adaptation to the parasitic lifestyle. Most of what we know about this phylum is based on the study of a subset of parasites of medical and veterinary importance, such as those causing malaria, cryptosporidiosis, neosporosis, and toxoplasmosis.

All Apicomplexa studied so far are characterized by complex life cycles, during which parasites transition through multiple life forms, often requiring intermediate hosts and vectorial transmission. This process involves multiple cell types and, consequently, differentiation and proliferation steps to adapt to the changing environment. Apicomplexa switch from intra- to extracellular environments; encysted slow-dividing forms, inaccessible to the immune system, to fast-dividing forms exposed to immunity; motile to non-motile. These transitions are all accompanied both by morphological and metabolic changes. The collection of articles in this Topic “Molecular Basis of Stage Conversion in Apicomplexan Parasites” reviews and updates current knowledge of the molecular mechanisms controlling stage conversion in some of the best-described Apicomplexa and presents new findings contributing to this field of study. It also integrates new insight into less-studied species, expanding our breadth of knowledge and understanding of the phylum as a whole.

*Plasmodium* spp. or *Toxoplasma gondii* parasites benefit from the improvement of a flexible genetic toolbox that permits in-depth dissection of stage transitions. Studies dissecting the regulation of gene expression and, ultimately, of stage transition have shown that a fine-tuned multilayer regulation of gene expression is necessary to control each transition. Understanding the dynamic morphological and metabolic changes at the structural and molecular level is paramount to developing effective prophylactic and treatment strategies as shown in Mévélec et al. In particular, stage-specific transcriptional regulation (involving epigenetic regulation, transcription factors, and chromatin organization) and translation repression are two mechanisms that shape the life cycle progression in both *Plasmodium. spp* as shown in Briquet et al. and Hollin and Le Roch and *T. gondii* (Sokol-Borelli et al.). In that respect, the recent characterization of the RNA Binding Protein UIS12 provides further evidence of the importance of post-transcriptional mechanisms in the control of *P. berghei* sexual stage development.

In addition to *Plasmodium* spp. and *T. gondii*, a number of other apicomplexan parasites are now amenable to genetic manipulation which has expanded our understanding of stage conversion in this phylum. The molecular basis of stage transition of coccidian parasites such as *Eimeria* spp. are now being investigated, demonstrating how metabolic pathways can be reshaped during transitions and contribute to the parasite’s adaptation to the environment as illustrated by Martorelli Di Genova and Knoll. Dissecting the biology of a wider selection of parasites from the phylum, such as *T. gondii’s* closest relatives *Hammondia hammondi* and *Neospora caninum*, is also important as it can also contribute to our understanding of the evolution of adaptations of these parasites to specific biological niches and hosts. For example, Sokol-Borelli et al. show high similarity between parasite genomes and the presence of identified key factors for transition in *T. gondii, which* does not necessarily indicate that these same actors are controlling stage transition in other parasites.

However, despite this progress, some parts of the complex life cycles of Apicomplexa are still not experimentally amenable. The emergence of new *in vitro* models is now allowing for access to large parts of the complex life cycle of these parasites. For example, dissecting the biology of the *T. gondii* sexual cycle may now be within reach, as illustrated by Tomasina and Francia. Such fast progress in the field would not be possible without a number of technological advancements, including in particular improvements in the use of next-generation sequencing technologies. This approach is now powerful enough to investigate Apicomplexa species or life cycle stages that are difficult to access. For example, from an extensive collection of *Cryptosporidium* transcriptomics data, Li et al. produced a new bioinformatic pipeline that identified almost 400 developmentally regulated long non-coding RNAs. These RNAs cover ~10% of the protein-coding genome and are upregulated in time points corresponding to gametocytogenesis, suggesting a role in sexual development. Next-generation sequencing also provides an in-depth characterization of the *T. gondii* transcriptome. Markus et al. mapped transcriptional start sites (TSS) of *T. gondii* with high resolution and stage-specific alternative TSS were identified for bradyzoite genes. *T. gondii* TSS were also analyzed in reference to recently published nucleosome data and found to be located unusually deep into the nucleosome, suggesting a role of chromatin architecture in stage conversion. It is clear that non-coding elements play important roles in differentiation and require further exploration.

In conclusion, the collection of articles in this Research Topic highlight the contribution of the study of underrepresented species and life stages, as well as the new improved technologies and model systems to current understanding of the molecular basis of stage transitions in Apicomplexa.

## Author Contributions

MG wrote the manuscript. MEF, KKM, and MCP edited the manuscript. All authors contributed to the article and approved the submitted version.

## Funding

MG would like to acknowledge the following funding agencies for their support: ANR, CNRS, Inserm, and Institut Pasteur de Lille (CPER-CTRL). MEF is funded by a Banco de Seguros del Estado installment grant, an ACIP grant from the RIIP, and the National Agency for Research and Innovation (ANII). MCP was supported by a Sir Henry Dale Fellowship jointly funded by the Wellcome Trust and the Royal Society (Grant Number 213469/Z/18/Z) and start-up funds from a Wellcome Trust Centre Award (Grant Number 203134/Z/16/Z). KKM is supported by the Wellcome Trust/Royal Society Sir Henry Dale award (202600/Z/16/Z).

## Conflict of Interest

The authors declare that the research was conducted in the absence of any commercial or financial relationships that could be construed as a potential conflict of interest.
